# Development and Validation of a Machine Learning–Based Prediction of Chronic Postsurgical Pain Following Total Hip and Knee Arthroplasty: An Exploratory Study

**DOI:** 10.1155/prm/5529087

**Published:** 2026-10-21

**Authors:** Yuan Cao, Yinghua Hu, Tianlun Gong, Zaijie Sun, Zijian Wang, Ximing Zhang

**Affiliations:** ^1^ Department of Orthopedics Xiangyang Central Hospital Affiliated Hospital of Hubei University of Arts and Science Xiangyang China xfszxyy.cn

## Abstract

**Introduction:**

Chronic postsurgical pain (CPSP) is a concern following total hip arthroplasty (THA) and total knee arthroplasty (TKA), substantially diminishing surgical benefits. We aimed to develop a machine learning (ML) prediction model for risk of developing CPSP.

**Methods:**

This cohort study included 383 patients recruited from a tertiary medical center between March 2022 and July 2024. Dataset was divided into a training set and a testing set. We used Lasso regression for feature selection and developed prediction models using six ML algorithms: logistic regression (LR), random forest (RF), extreme gradient boosting (XGBOOST), support vector machine (SVM), multilayer perceptron (MLP), and k‐nearest neighbor (KNN). Model performance was evaluated using metrics such as receiver operator characteristic (ROC) curve and precision–recall (PR) curves, accuracy, sensitivity, and specificity. The optimal model was deployed on a web application using Shiny, and Shapley additive explanations (SHAP) analysis was used to interpret model predictions.

**Results:**

Among the 383 eligible patients, the median age was 67.4 years, and 58.0% were women. The incidence of CPSP in TKA and THA was 26.0% and 12.7% respectively. Four predictors were selected: preoperative pain catastrophizing scale (PRO‐PCS), preoperative Tampa Scale for Kinesiophobia (PRO‐TSK), preoperative numerical rating scale (PRO‐NRS) at rest, and time to first ambulation. In the testing set, the RF model demonstrated the best performance with ROC‐AUC of 0.872 and PR‐AUC of 0.773. The accuracy, sensitivity, and specificity were 0.90, 0.77, and 0.94, respectively.

**Conclusion:**

This study developed a clinically applicable ML model for CPSP prediction for THA, while the CPSP prediction for TKA was only conducted for exploration and still requires further verification.

## 1. Introduction

Arthroplasty, such as total hip arthroplasty (THA) and total knee arthroplasty (TKA), has become a routine and effective treatment for end‐stage joint diseases, significantly improving patients’ quality of life by alleviating pain and enhancing joint function [1]. However, approximately 10%–30% of patients suffers from chronic postsurgical pain (CPSP), which can persist for months or even years after surgery [2–6]. CPSP not only diminishes the benefits of joint replacement but also imposes a substantial burden on patients’ physical and mental well‐being, as well as on healthcare resources [3, 7]. Early identification of patients at high risk of developing chronic pain after joint replacement is crucial for optimizing surgical decision‐making, facilitating preoperative patient counseling, and enabling targeted interventions to improve postoperative outcomes [6].

Numerous studies have attempted to identify risk factors for CPSP and develop relevant prediction models. These factors encompass a wide range of variables, including preoperative pain intensity and duration, psychological status such as anxiety, depression, and pain catastrophizing, surgical techniques, complications, and patient demographics like age and gender [2, 8–22]. However, the association between these risk factors and the development of chronic pain is intricate and often nonlinear. Traditional statistical methods, such as logistic regression (LR), may fail to capture the complex interactions among multiple variables. Moreover, the prediction models developed alongside these factors lack feasible visualization, making clinical application difficult. In recent years, machine learning (ML) techniques have emerged as powerful tools in medical research. They have demonstrated remarkable capabilities in handling large and complex datasets, uncovering hidden patterns, and generating accurate predictions. Its shiny‐based web calculators are now widely used in various medical scenarios, such as predicting surgical outcomes, diagnosing diseases, and creating personalized treatment plans [23–26].

In this study, we aimed to develop and validate a ML‐based prediction model for estimating the risk of CPSP. Utilizing a dataset that encompasses patient demographics, preoperative assessments, surgical details, and postoperative follow‐up information, we identified key predictors of CPSP and developed a predictive tool to support clinical risk assessment at the individual level. To enhance the interpretability and clinical utility of the model, we applied Shapley additive explanations (SHAP) for explaining individual predictions. The resulting model is intended to assist clinicians in identifying patients at high risk of CPSP, thereby facilitating early intervention and personalized management.

## 2. Methods

### 2.1. Experimental Design and Participants

The prospective cohort study followed the tripod guidelines for developing a multivariable prediction model targeting patients undergoing THA or TKA and reporting guidelines for artificial intelligence use in orthopedic surgery research [27, 28]. The patients were recruited from Xiangyang Central Hospital between March 2022 and July 2024. We divided the dataset into a training set, consisting of 287 patients from March 2022 to September 2023, and a testing set, involving 96 patients from October 2023 to July 2024. Using the training set, we performed feature selection and model development. The testing set was then utilized for model performance evaluation and comparison, leading to the identification of the optimal model and its visualization. All study steps are outlined in a flowchart (Figure 1). Written consent was obtained from all participants, and all clinical data were anonymized. The study protocol was approved by the medical ethical committee of the participating center (ethical batch: No. 2022‐004) and conducted in accordance with the provisions of the Declaration of Helsinki concerning medical research involving human subjects.

**FIGURE 1:**
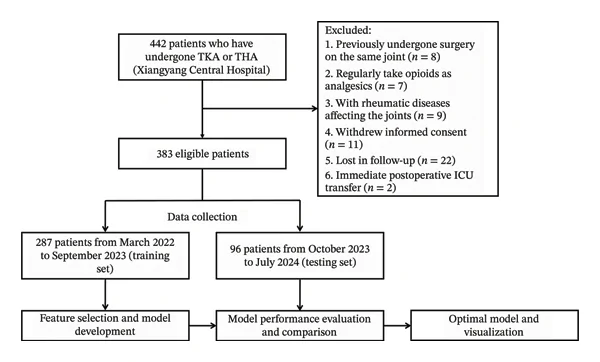
Flowchart of participant enrollment and the study design.

Patients meeting the following criteria were enrolled: (1) aged ≥ 65 years, (2) American Society of Anesthesiologists (ASA) Physical Status Classification System I–III, (3) scheduled for total knee or hip arthroplasty, (4) able to complete self‐assessment questionnaires, and (5) provided written informed consent. Patients meeting the following criteria were excluded: (1) previously undergone surgery on the same joint, (2) regularly take opioids as analgesics, (3) with rheumatic diseases affecting the joints or other conditions that may cause joint pain (such as lumbar radiculopathy or peripheral neuropathy), (4) withdrew informed consent, (5) lost in follow‐up, and (6) immediate postoperative ICU transfer.

### 2.2. Data Collection

Preoperative variables, perioperative records, and postoperative outcomes were extracted from the electronic medical record and questionnaires, guided by previous studies [10, 18, 20, 22, 29–31]. Due to the prospective study design and standardized data collection protocol, the final predictors selected by the least absolute shrinkage and selection operator (Lasso) regression had no missing values, allowing for a complete case analysis without the need for imputation. These were grouped into four domains: (1) demographics: age, gender, body mass index (BMI), educational level, employment status, living status, smoking, and alcohol drinking; (2) medical history: hypertension, diabetes, cardiovascular or cerebrovascular disease, insomnia, surgical history among surgical site, and current use of analgesics; (3) perioperative records: type of arthroplasty, ASA grade, operation duration, anesthesia duration, type of anesthesia, intraoperative blood loss, length of stay, time to first ambulation, and postoperative use of analgesics or patient‐controlled analgesia (PCA); (4) questionnaires: preoperative numerical rating scale (PRO‐NRS) at rest and during activity, preoperative 14‐item Hamilton anxiety rating scale (PRO‐HAMA‐14) and preoperative 17‐item Hamilton depression rating scale (PRO‐HAMD‐17), preoperative pain catastrophizing scale (PRO‐PCS), preoperative Tampa Scale for Kinesiophobia (PRO‐TSK), and postoperative numerical rating scale (POO‐NRS) at rest and during activity.

To ensure diagnostic accuracy, all patients underwent at least one scheduled in‐person follow‐up visit after surgery, which included wound inspection and suture removal, structured interview, physical examination, and weight‐bearing radiographs of the operated joint. In addition, patients were explicitly instructed to return for an unscheduled emergency or specialist visit at any time if they developed persistent pain, worsening swelling, fever, sudden restriction of movement, prosthetic noise, or limb shortening. Telephone follow‐ups were conducted only as a supplementary means for patients who were unable to attend in‐person visits in a timely manner and were not used as the sole basis for outcome classification.

All patients with persistent pain at the 3‐month, 6‐month, or 12‐month follow‐up underwent orthopedic physical examination by an attending orthopedic surgeon to assess for signs of infection (warmth, erythema, effusion), instability, malalignment, or patellofemoral problems (in TKA).

Radiographic assessment (X‐rays, CT, or MRI) was selectively performed according to patient symptoms, especially in those reporting persistent pain. Particular attention was paid to prosthetic loosening, osteolysis, and alignment.

If infection was suspected based on clinical signs or laboratory findings (elevated CRP/ESR), further work‐up including joint aspiration was performed.

CPSP was diagnosed only after these alternative local causes were excluded.

### 2.3. Definition

#### 2.3.1. CPSP

CPSP was defined as clinically relevant pain (NRS score ≥ 4) located at the operated joint or its referral zones that develops or increases, during any of the three postoperative follow‐up assessments (3, 6, or 12 months). Persistence was confirmed if the patient reported a pain duration of ≥ 3 months at any of these follow‐up points. All cases of reported pain were carefully reviewed to exclude pain attributable to other causes, such as infection, implant failure, or unrelated disease [32, 33].

The primary outcome time point for classification was 3, 6, and 12 months postoperatively. CPSP was considered present if the patient reported continuous pain more than 3 months (NRS score ≥ 4 at the operated joint or its referral zones) at any of the follow‐up time points [34–37].

#### 2.3.2. NRS

Current pain intensity was captured with an 11‐point NRS, where 0 represents “no pain” and 10 “worst imaginable pain.” Patients were asked to report their average pain over the preceding 24 h. Scores ≥ 4 denote moderate‐to‐severe pain [34–37].

#### 2.3.3. HAMA‐14

Anxiety symptoms were measured with the HAMA‐14. Each item is scored 0–4, producing a total range of 0–56. Higher scores reflect greater anxiety severity; clinically significant anxiety was defined as a total score ≥ 14 [38].

#### 2.3.4. HAMD‐17

Depressive symptoms were assessed using the HAMD‐17. The total score ranges from 0 to 52, with higher scores indicating more severe depression. A score ≥ 17 was used to denote clinically relevant depressive symptoms [39].

#### 2.3.5. PCS

The Chinese version of the PCS evaluates catastrophic thinking related to pain and contains 13 items rated 0–4. The summary score ranges from 0 to 52. A total ≥ 38 identifies high pain catastrophizing [40].

#### 2.3.6. TSK

Fear of movement was quantified with the Chinese version of the 17‐item TSK. Each item is scored 1–4, producing a total range of 17–68. A score ≥ 37 indicates clinically relevant kinesiophobia [41].

#### 2.3.7. Time to First Ambulation

Time to first ambulation refers to the interval from the end of surgery to the first successful attempt at independent or assisted walking over 5 m, completed under clinical supervision without adverse events (e.g., falls, hemodynamic instability) and initiated within the earliest safe window as permitted by surgical protocols and rehabilitation guidelines [42].

### 2.4. Predictors

We used the Lasso regression to select, from candidate variables (Table 1), the subset most predictive of CPSP. By shrinking redundant coefficients to zero, Lasso curbed multicollinearity and yielded a compact predictor set for subsequent model development (Figure 2) [43].

**TABLE 1:** Clinical characteristics associated with CPSP among patients undergoing arthroplasty.

Variables	Training set (*n* = 287)		*p*	Testing set (96)		*p*
	Non‐CPSP (*n* = 228)	CPSP (*n* = 59)		Non‐CPSP (*n* = 70)	CPSP (*n* = 26)	
Gender			0.206			0.739
Men	102 (44.7%)	21 (35.6%)		27 (38.6%)	11 (42.3%)	
Women	126 (55.3%)	38 (64.4%)		43 (61.4%)	15 (57.7%)	
Age (y)	67.3 ± 6.64	67.6 ± 6.52	0.778	70.0 (65.3, 72.0)	66.5 (64.0, 70.0)	0.141
BMI (kg/m^2^)	23.0 (22.0, 25.0)	24.0 (22.0, 25.0)	0.087	23.1 ± 2.63	24.1 ± 2.36	0.088
Educational level			0.091			0.630
Middle school or below	117 (51.3%)	23 (39.0%)		42 (60.0%)	17 (65.4%)	
High school or above	111 (48.7%)	36 (61.0%)		28 (40.0%)	9 (34.6%)	
Employment status			0.369			**0.030**
Employed	22 (9.6%)	4 (6.8%)		16 (22.9%)	2 (7.7%)	
Unemployed	152 (66.7%)	36 (61.0%)		45 (64.3%)	15 (57.7%)	
Retired	54 (23.7%)	19 (32.2%)		9 (12.9%)	9 (34.6%)	
Live alone	29 (12.7%)	11 (18.6%)	0.242	8 (11.4%)	6 (23.1%)	0.194
Smoking	24 (10.5%)	6 (10.2%)	0.936	9 (12.9%)	8 (30.8%)	0.068
Alcohol drinking	35 (15.4%)	8 (13.6%)	0.731	9 (12.9%)	3 (11.5%)	> 0.999
Medical history						
Hypertension	99 (43.4%)	31 (52.5%)	0.210	35 (50.0%)	16 (61.5%)	0.314
Diabetes	30 (13.2%)	7 (11.9%)	0.792	12 (17.1%)	3 (11.5%)	0.753
Cardiovascular and cerebrovascular diseases	47 (20.6%)	13 (22.0%)	0.811	14 (20.0%)	0 (0.0%)	**0.010**
Insomnia	89 (39.0%)	25 (42.4%)	0.640	33 (47.1%)	14 (53.8%)	0.559
Use of analgesics	37 (16.2%)	12 (20.3%)	0.454	7 (10.0%)	5 (19.2%)	0.297
PRO‐HAMA‐14	16.3 ± 5.45	18.4 ± 5.55	**0.012**	16.1 ± 4.6	18.3 ± 4.4	**0.035**
PRO‐HAMD‐17	13.8 ± 4.43	17.5 ± 5.23	**< 0.001**	14.8 ± 4.7	18.6 ± 6.7	**0.012**
PRO‐PCS	18.3 ± 6.18	29.6 ± 8.10	**< 0.001**	18.1 ± 6.30	29.4 ± 6.93	**< 0.001**
PRO‐TSK	29.8 ± 7.27	43.3 ± 10.51	**< 0.001**	30.2 ± 7.37	41.7 ± 9.63	**< 0.001**
PRO‐NRS (rest)	2.1 ± 1.55	3.1 ± 1.34	**< 0.001**	2.0 (1.0, 3.0)	3.0 (2.0, 5.0)	**0.021**
PRO‐NRS (activity)	5.4 ± 2.44	5.9 ± 2.04	0.135	5.0 (3.0, 7.0)	6.0 (5.0, 8.0)	**0.035**
Operation			**0.017**			0.147
TKA	153 (67.1%)	49 (83.1%)		49 (70.0%)	22 (84.6%)	
THA	75 (32.9%)	10 (16.9%)		21 (30.0%)	4 (15.4%)	
Previous surgical history among surgical site	34 (14.9%)	10 (16.9%)	0.699	14 (20.0%)	3 (11.5%)	0.548
ASA classification			0.307			0.281
Grade I	45 (19.7%)	17 (28.8%)		20 (28.6%)	8 (30.8%)	
Grade II	158 (69.3%)	37 (62.7%)		46 (65.7%)	14 (53.8%)	
Grade III	25 (11.0%)	5 (8.5%)		4 (5.7%)	4 (15.4%)	
Operation duration	105.8 ± 39.16	110.6 ± 36.01	0.364	102.9 ± 33.88	118.8 ± 39.94	0.077
Anesthesia duration	125.7 ± 38.25	129.6 ± 47.76	0.565	138.7 ± 45.28	124.0 ± 44.58	0.093
Anesthesia			0.950			0.566
General anesthesia	185 (81.1%)	49 (83.1%)		52 (74.3%)	22 (84.6%)	
Intraspinal anesthesia	7 (3.1%)	2 (3.4%)		3 (4.3%)	0 (0.0%)	
Combined with PNB anesthesia	36 (15.8%)	8 (13.6%)		15 (21.4%)	4 (15.4%)	
Intraoperative blood loss (mL)	129.6 ± 48.12	116.4 ± 41.89	**0.039**	123.0 (87.3, 148.0)	127.0 (86.3, 167.0)	0.531
Postoperative use of analgesics	174 (76.3%)	46 (78.0%)	0.789	51 (72.9%)	21 (80.8%)	0.426
Postoperative use of PCA pump	140 (61.4%)	39 (66.1%)	0.507	51 (72.9%)	19 (73.1%)	0.983
Length of stay (d)	14.5 ± 3.52	14.4 ± 3.07	0.821	14.5 ± 3.14	15.0 ± 4.19	0.534
Time to first ambulation (d)	2.2 ± 1.21	3.9 ± 0.68	**< 0.001**	2.0 (1.0, 3.0)	4.0 (3.3, 4.0)	**< 0.001**
POO‐NRS (rest)	2.4 ± 1.34	2.9 ± 1.52	**0.022**	3.0 (2.0, 4.0)	3.0 (2.0, 4.0)	0.326
POO‐NRS (activity)	5.1 ± 2.15	5.9 ± 2.06	**0.008**	5.0 (4.0, 5.8)	6.5 (5.0, 7.8)	**< 0.001**

*Note:* CPSP, chronic postsurgical pain; PRO, preoperative; HAMA‐14, 14‐item Hamilton anxiety rating scale; HAMD‐17, 17‐item Hamilton depression rating scale; POO, postoperative; median (Q1, Q3); *n* (%); mean ± SD. Bold font indicates significant results.

Abbreviations: ASA, American Society of Anesthesiologists; BMI, body mass index; NRS, numerical rating scale; PCA, patient‐controlled analgesia; PCS, pain catastrophizing scale; PNB, peripheral nerve block; THA, total hip arthroplasty; TKA, total knee arthroplasty; TSK, Tampa Scale for Kinesiophobia.

**FIGURE 2:**
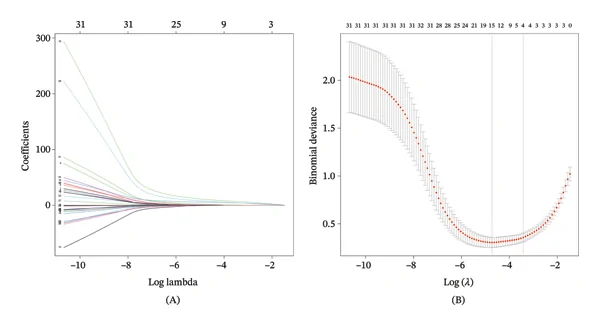
Least absolute shrinkage and selection operator regression results. (A) Coefficient paths of variables at different log (lambda) values. (B) Binomial deviance across log (lambda) values, highlighting the optimal lambda selection.

### 2.5. Sample Size Calculation

Sample size calculation was performed with the pmsampsize package in R (4.3.3). Based on previous relevant study, the incidence of CPSP is 20.9% after TKA and 7.5% after THA, with a TKA:THA ratio of 2:1; the pooled expected incidence was, therefore, 16.4%. Targeting 3–5 candidate predictors, a C‐statistic of 0.80, and a shrinkage factor of 0.90, the package indicated a required sample size of 211–256 participants. At a 7:3 split, the testing set required 64–77 participants.

### 2.6. Model Development

The model was validated using a temporally split, held‐out testing set, which was not used in any part of model training or feature selection. In addition, to ensure robustness and mitigate overfitting, fivefold cross‐validation was performed on the training set during the hyperparameter tuning phase. The performance metrics were evaluated based on the independent testing set. Six ML algorithms were used to develop prediction models for CPSP following arthroplasty: The six ML algorithms were selected to represent a diverse range of modeling approaches: LR as a robust linear baseline; random forest (RF) and extreme gradient boosting (XGBOOST) as powerful ensemble methods capable of capturing complex, nonlinear interactions; support vector machine (SVM) for its effectiveness in high‐dimensional spaces; multilayer perceptron (MLP) as a representative neural network; and k‐nearest neighbor (KNN) as an instance‐based learner. This selection allows for a comprehensive comparison to identify the most suitable algorithm for the data structure at hand. The prediction performance and clinical application value of these models were comprehensively assessed through metrics such as accuracy, sensitivity, specificity, positive predictive value (PPV), negative predictive value (NPV), f‐measure, kappa, receiver operator characteristic (ROC), and precision–recall (PR) curves. The cutoff value is calculated based on the ROC curve. The optimal model after predictive performance comparison was deployed on a web application using Shiny for easy application.

The feature importance of the models was analyzed using SHAP, and the model interpretability was demonstrated through SHAP feature importance ranking graphs and SHAP summary plots. The study employed the R package tidymodels (1.2.0) for training and comparing the different ML models.

### 2.7. Statistical Analysis

Shapiro–Wilk tests were conducted to evaluate the normality of continuous variables. Normally distributed continuous variables were presented as mean ± SD and compared using independent samples *t*‐tests, while nonnormally distributed variables were reported as median (Q1, Q3) and compared with Mann–Whitney *U* tests. Categorical variables were expressed as *n* (%) and analyzed using Fisher’s exact test or chi‐square test depending on the sample size. A threshold of *p* < 0.05 was established for statistical significance. All statistical analyses were performed using R Version 4.3.3.

## 3. Results

### 3.1. Demographic and Clinical Characteristics

Initially, 442 patients who had undergone TKA or THA were recruited from a tertiary medical center. Finally, 383 eligible patients were included in the study. Recruitment of patients was conducted from March 2022 to September 2023 for the training set, comprising 287 patients, and from October 2023 to July 2024 for the testing set, comprising 96 patients (Figure 1). The median age was 67.4 years, and the majority of patients were women, accounting for 58.0%. The median BMI was 23.4. The most common operation was TKA, with 71.3%. After the end of the 1‐year follow‐up period, a total of 85 patients (85/383, 22.2%) developed CPSP, including 71 patients (71/273, 26.0%) who underwent TKA and 14 patients (14/110, 12,7%) who underwent THA. Table 1 presents the demographic and clinical characteristics of both the non‐CPSP and CPSP groups. Patients with CPSP had a significantly higher PRO‐HAMA‐14 score, PRO‐HAMD‐17 score, PRO‐PCS score, PRO‐TSK score, and PRO‐NRS (rest) score compared to those without CPSP.

### 3.2. Model Performance

Following Lasso regression for variable selection, four predictors were identified: PRO‐PCS, PRO‐TSK, PRO‐NRS (rest), and time to first ambulation (Figure 2).

Table 2 summarizes the models’ prediction performances. On the testing set, the RF model achieved the highest ROC‐AUC of 0.872 and the highest PR‐AUC of 0.7731, while LR achieved 0.8434 and 0.7102, respectively. The RF model achieved 0.90 accuracy, 0.77 sensitivity, and 0.94 specificity in the testing set (Table 2 and Figure 3).

**TABLE 2:** Prediction performance of ML models in the testing set.

Model	Accuracy	Sensitivity	Specificity	PPV	NPV	ROC‐AUC
LR	0.90	0.81	0.93	0.81	0.93	0.84
RF	0.90	0.77	0.94	0.83	0.92	0.87
XGBOOST	0.84	0.69	0.90	0.72	0.89	0.85
SVM	0.84	0.81	0.86	0.68	0.92	0.85
MLP	0.86	0.69	0.93	0.78	0.89	0.84
KNN	0.84	0.69	0.90	0.72	0.89	0.85

*Note:* XGBOOST, eXtreme gradient boosting; MLP, multilayer perceptron.

Abbreviations: AUC, area under the curve; KNN, K‐nearest neighbor; LR, logistic regression; NPV, negative predictive value; PPV, positive predictive value; RF, random forest; ROC, receiver operator characteristic; SVM, support vector machine.

**FIGURE 3:**
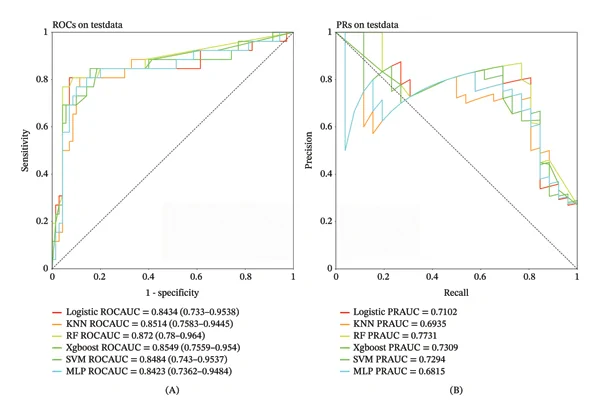
Model performance evaluation. (A) Receiver operating characteristic curves comparing different models’ performance in the testing set, with area under the curve values indicated for each model. (B) Precision–recall curves comparing different models’ performance in the testing set, with area under the curve values indicated for each model.

Given the differing incidence of CPSP between TKA and THA, exploratory subgroup analyses were conducted. The RF model for TKA (*n* = 71) and THA (*n* = 25) in the testing set retained good discrimination (ROC‐AUC = 0.8752 and 0.85), while the model for THA (*n* = 25) showed lower performance on the PR‐AUC (AUC = 0.6964) likely due to the smaller sample size and fewer events (*n* = 5).

TKA‐only sensitivity analysis: First, repeated feature selection was performed using Lasso regression in the TKA cohort, confirming the same four key predictors (PRO‐PCS, PRO‐TSK, PRO‐NRS at rest, and time to first ambulation). Second, model discrimination, calibration curve, and confusion matrix of the RF model in the TKA‐only subset were 0.8771 [0.7703–0.984]) of ROC‐AUC and 0.7853 [0.5828–0.9528]) of PR‐AUC. Third, feature importance ranking in TKA was consistent with prior results.

### 3.3. SHAP Explanation

Figure 4A presents the feature importance rankings derived from the RF model. The analysis identified four key predictors that significantly influence the development of CPSP. The most important feature was the number of days to first ambulation postoperatively, followed by PRO‐PCS, PRO‐TSK, and PRO‐NRS (rest) scores. These features were determined based on the one‐minus‐AUC loss after permutations, indicating their relative importance in the model.

**FIGURE 4:**
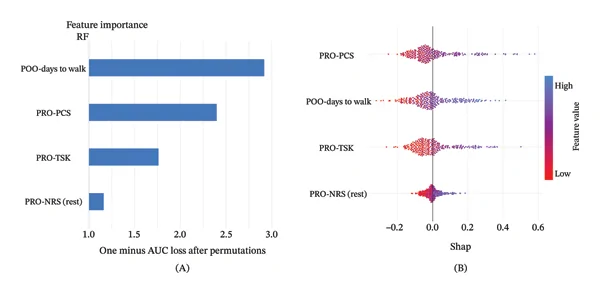
Feature importance and Shapley additive explanations analysis. (A) Random forest–based feature importance ranking, showing key predictors of chronic postsurgical pain based on one‐minus‐area under the curve loss after permutations. (B) Shapley additive explanations summary plot illustrating each feature’s impact on model predictions.

Figure 4B illustrates the SHAP summary plot, which provides a detailed view of the impact of each feature on the model’s predictions. The SHAP values represent the contribution of each feature to the prediction for each individual patient. The color gradient indicates the value of the feature, with blue representing higher values and red representing lower values. The spread of the points along the SHAP value axis shows the positive or negative contributions each feature makes to the prediction.

Figures 5 demonstrates the prediction interface for the RF model (Figure 5A,B). The interface enables users to input specific patient data and to obtain a probability prediction for development of CPSP. The SHAP contribution plots (Figure 5C,D) further break down the prediction by showing the contribution of each feature to the final prediction score. The prediction interface is accessible online (https://xxxhospital.shinyapps.io/CPSP/). Figure 6 demonstrates the comprehensive algorithm tree to enhance clinical applicability.

**FIGURE 5:**
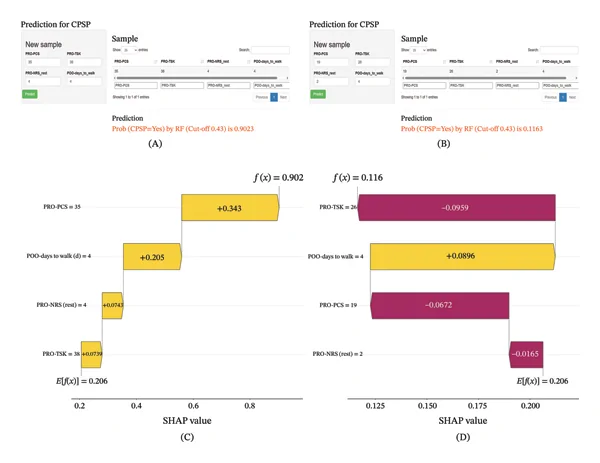
Chronic postsurgical pain prediction interface and Shapley additive explanations analysis contribution plots. (A) Prediction interface for a sample with a high probability of chronic postsurgical pain (0.9023), showing input features and predicted risk. (B) Prediction interface for a sample with a low probability of chronic postsurgical pain (0.1163), showing input features and predicted risk. (C) Contribution plot for the high‐risk sample, highlighting feature contributions to the prediction. (D) Contribution plot for the low‐risk sample, highlighting feature contributions to the prediction.

**FIGURE 6:**
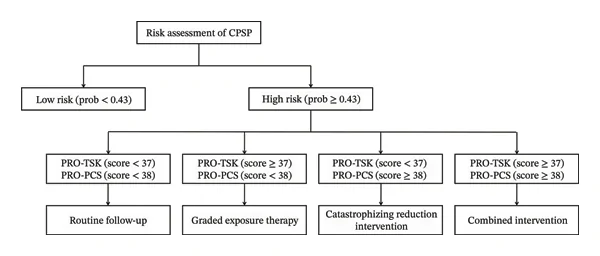
Conceptual framework for risk‐stratified intervention considerations. A hypothesis‐generating algorithm that requires prospective evaluation of feasibility, cost‐effectiveness, and patient adherence before clinical implementation.

## 4. Discussion

CPSP remains a significant challenge following THA and TKA, affecting 10%–30% of patients [5, 22, 44]. This study developed and validated a ML‐based prediction model for CPSP risk using prospectively collected data from 383 patients. Key findings include the following: first, CPSP incidence was 26.0% in patients undergoing TKA compared to 12.7% in patients undergoing THA during the 1‐year follow‐up period. Second, higher PRO‐PCS, PRO‐TSK, PRO‐NRS (rest) scores and longer time to first ambulation were key predictors of CPSP. Third, among ML algorithms evaluated, the RF model demonstrated superior discrimination.

The observed CPSP incidence after TKA and THA aligns with epidemiological reports from large cohort studies [22, 29, 44]. The significantly higher risk in TKA patients (RR = 2.05, 95% CI: 1.21–3.48) corroborates biomechanical evidence: the knee’s complex proprioceptive network and higher functional demands render it more susceptible to persistent nociceptive sensitization compared to the hip.

We acknowledge that the pooled analysis was conducted primarily for exploratory purposes in THA. Although we included both THA and TKA patients in the dataset, the main analysis was restricted to TKA, for which we had adequate sample size and event numbers. The THA data are presented only as exploratory, and the model should not be interpreted as equally validated for THA. The four LASSO‐selected predictors were identical when feature selection was performed separately in the TKA and THA subgroups, but the small THA sample (*n* = 110, CPSP events = 14) precludes reliable independent model development or validation.

Previous studies have explored CPSP risk factors and prediction models. Our study aligns with findings that psychological factors (e.g., anxiety, depression, pain catastrophizing) and postoperative functional recovery (e.g., time to first ambulation) are crucial predictors. For instance, a study by Kris et al. emphasized the role of PRO‐PCS in predicting CPSP following TKA and function [9]. Similarly, our results showed PRO‐PCS and PRO‐TSK to be key predictors. Additionally, our identification of time to first ambulation as a significant predictor echoes research highlighting postoperative functional recovery’s impact on CPSP development. It is emphasized that the predictive power of time to first ambulation in our model likely reflects its role as a surrogate marker for the severity of acute postoperative pain and the subsequent risk of central sensitization, rather than being a direct causal factor independent of pain intensity. The interpretation of “time to first ambulation” as a predictor of CPSP is nuanced. While delayed ambulation may reflect severe acute postoperative pain, a known risk factor for chronic pain, it can also result from nonpain factors such as postoperative nausea and vomiting, orthostatic intolerance, sedation, or logistical delays. If the delayed ambulation is caused by factors other than pain, then it does not apply to this model.

To translate our findings into actionable clinical guidance, we developed a decision algorithm (Figure 6) that stratifies patients by risk level and suggests targeted interventions based on their prominent risk factors (e.g., graded exposure therapy for high PRO‐TSK, cognitive‐behavioral approaches for high PRO‐PCS). We recognize that integrating cognitive‐behavioral or graded exposure therapy into routine arthroplasty pathways faces substantial barriers, including limited access to specialized behavioral health providers and high patient burden. The proposed algorithm in Figure 6 should, therefore, be viewed as a conceptual risk‐stratification framework to guide future implementation research, rather than an immediately actionable clinical pathway.

Although LR achieved comparable performance in this specific dataset with only four predictors, the RF model was ultimately chosen due to its superior discrimination (ROC‐AUC 0.872 vs. 0.835 for LR, PR‐AUC 0.8434 vs. 0.7102), better handling of potential nonlinear relationships, and the ability to generate interpretable SHAP plots for individual risk prediction [23–26]. Nevertheless, we acknowledge that for parsimonious models with few predictors, conventional regression remains a viable and simpler alternative.

However, limitations exist. First, the study was conducted at a single center, which may limit generalizability to other populations. Future multicenter studies are needed to validate the model’s performance across diverse settings. Second, routine postoperative imaging was not performed for all patients—only for those reporting persistent pain. Third, telephone follow‐ups were used as a supplementary means for patients unable to attend in‐person visits, which may have limited the accuracy of pain characterization. Fourth, despite our systematic exclusion efforts, residual misclassification of CPSP cannot be entirely ruled out. We, therefore, characterize the outcome as “confirmed CPSP after systematic clinical and radiographic exclusion” rather than implying definitive diagnosis in every case.

## 5. Conclusion

This study developed a clinically applicable ML model for CPSP prediction for THA, while the CPSP prediction for TKA was only conducted for exploration and still requires further verification.

## Author Contributions

Yuan Cao: writing–original draft. Yinghua Hu: methodology, formal analysis, data curation. Tianlun Gong: validation, supervision. Zaijie Sun: investigation. Zijian Wang: project administration, methodology, data curation, supervision. Ximing Zhang: supervision, writing–review and editing.

## Funding

No funding was received for this manuscript.

## Conflicts of Interest

The authors declare no conflicts of interest.

## Data Availability

The data that support the findings of this study are available from the corresponding author upon reasonable request.
